# Multiple Inter-Kingdom Horizontal Gene Transfers in the Evolution of the Phosphoenolpyruvate Carboxylase Gene Family

**DOI:** 10.1371/journal.pone.0051159

**Published:** 2012-12-12

**Authors:** Yingmei Peng, Jing Cai, Wen Wang, Bing Su

**Affiliations:** 1 State Key Laboratory of Genetic Resources and Evolution, Kunming Institute of Zoology, Chinese Academy of Sciences, Kunming, PR China; 2 Shenzhen Key Laboratory for Orchid Conservation and Utilization, National Orchid Conservation Center of China and Orchid Conservation and Research Center of Shenzhen, Shenzhen, China; 3 Center for Biotechnology and BioMedicine, Graduate School at Shenzhen, Tsinghua University, Shenzhen, China; 4 University of Chinese Academy of Sciences, Beijing, PR China; University of Melbourne, Australia

## Abstract

Pepcase is a gene encoding phosphoenolpyruvate carboxylase that exists in bacteria, archaea and plants,playing an important role in plant metabolism and development. Most plants have two or more pepcase genes belonging to two gene sub-families, while only one gene exists in other organisms. Previous research categorized one plant pepcase gene as plant-type pepcase (PTPC) while the other as bacteria-type pepcase (BTPC) because of its similarity with the pepcase gene found in bacteria. Phylogenetic reconstruction showed that PTPC is the ancestral lineage of plant pepcase, and that all bacteria, protistpepcase and BTPC in plants are derived from a lineage of pepcase closely related with PTPC in algae. However, their phylogeny contradicts the species tree and traditional chronology of organism evolution. Because the diversification of bacteria occurred much earlier than the origin of plants, presumably all bacterialpepcase derived from the ancestral PTPC of algal plants after divergingfrom the ancestor of vascular plant PTPC. To solve this contradiction, we reconstructed the phylogeny of pepcase gene family. Our result showed that both PTPC and BTPC are derived from an ancestral lineage of gamma-proteobacteriapepcases, possibly via an ancient inter-kingdom horizontal gene transfer (HGT) from bacteria to the eukaryotic common ancestor of plants, protists and cellular slime mold. Our phylogenetic analysis also found 48other pepcase genes originated from inter-kingdom HGTs. These results imply that inter-kingdom HGTs played important roles in the evolution of the pepcase gene family and furthermore that HGTsare a more frequent evolutionary event than previouslythought.

## Introduction

Following wide acceptance of Darwin's theory of evolution, the tree of life became a well accepted representation of the evolutionary relationships among organisms. Recent findings of the horizontal gene transfer (HGT) in the genomes of many species [1], [2], [3], [4], [5], [6] strongly challenge this certainty. HGT, though, is still thought as rare event and genes that originated from HGT account for a tiny proportion in each genome, while vertical descent of genes remains the major mechanism of evolution.Moreover, all HGT genes are treated as noise when species phylogeny is constructed. Here, for the first time, we found 48 members from well supported inter-kingdom HGT in a single gene family coding phosphoenolpyruvate carboxylase. This case demonstratesthe means by which the evolution of a single gene family can form a complex web via horizontal gene transfer, and likewise suggests that the previously ignored contribution of HGT to the evolution pattern would strongly enhance our understanding of the evolution as a tree of life to more rich and diversified web of life that revealsthe unexpected complexity of evolution.

Phosphoenolpyruvate carboxylase (PEPC) is an important enzyme that catalyzes the carboxylation reaction of phosphoenolpyruvate into oxalacetate, which is then used by the citric cycle. This reaction is also used by C4 and crassulacean acid metabolic pathway and is an important step to store and concentrate carbon dioxide for photosynthesis. In 2003, Sanchez and Cejudo found a PEPC gene in Arabidopsis and rice with close homologs with PEPCs in bacteria [7]. Since then, the plant PEPC gene family has been categorized in to plant-type (PTPC) and bacteria-type (BTPC) subfamilies. Despite this organization, the actual evolution of the whole gene family has not been discussed in any detail. Only O'Leary et al.'s [8] recent review included a constructed phylogeny of PEPC gene family including members from Archaea, Bacteria, protists and plants. In this tree, the BTPC were clustered with bacteria PEPCs forming a clade as a sister group of protist PEPC. This phylogeny showed that the ancestor of all bacteria PEPCs, protists PEPCs and BTPCs originated from a duplication event in the lineage of PTPC to algae after its divergence with vascular plant PTPCs. This gene phylogeny has many inconsistencies with the accepted species tree constructed by multiple gene analysis and can only be explained by multiple gene transfer from the common ancestor of all BTPC, protists PEPC and bacteria PEPC to the ancestor of protists and bacteria. There is one remaining problem: the diversification of bacteria is a very ancient event,predating the divergence between algae and vascular plants. In theory, the duplicated copy of the ancestral PTPC which postdates the divergence of vascular and algal plant PTPC can by no means be transferred to the ancestors of the bacteria. Reconciliation between the gene tree and species tree is then almost impossible. This phylogeny must be reconsidered with caution.

We searched the GenBank and UniProt to explore the entire range of existent PEPC genes in all organisms sequenced in the database. We identified possible inter-kingdom HGT candidates in PEPC family, and constructed the gene family phylogeny with genes from representative taxa and those identified inter-kingdom HGT candidates in order to clarify the evolution of this gene family and validate the suspected inter-kingdom HGT events.

## Results and Discussion

We searched the GenBank by BLASTP and tBLASTn using PEPCs as a query and found that PEPC is a widely spread gene in archaea, prokaryotes and eukaryotes. In eukaryotes, PEPC exists mostly in plants, protists and slime mold. Only two hits were found in animals:The first was found in the genome of the black-legged tick, *Ixodesscapularis*. The 164-amino-acid fragment on the C-terminus of a 193-amino-acid protein(gene ID: 8031581) has 100% identity with pepcase from an alpha-proteobacterium, *Rhodobacterales bacterium* HTCC2255. Because this peptide is very short and possibly non-functional, it may be the relic of a recent unsuccessful horizontal gene transfer. The second was found in the genome of platypus, *Ornithorhynchusanatinus*. This is a peptide of 374 amino-acid (gene ID: 345310721) coded on a short contig of 1,614 base pair in the genome assembly. This gene has its closest homolog (e value, 3e-98) in a parasite, *Babesiabovis*. This may be a result of gene transfer from the parasite to the host, but we cannot exclude the possibility of parasitic genome pollution during genomic DNA preparation of the sequencing project.

We confirmed our suspicion of parasite contamination after reviewing the gene family information in Pfam database, in which we found two PEPC gene families, PEPcase (PF00311) and PEPcase_2 (PF14010). PEPcase is distributed in bacteria and eukaryotes including plants, protists and slime mold, while PEPcase_2 is mainly distributed in Archaea. However, there are also members within the two gene families whose taxonomy positions are incongruent with the main distribution, potentially due to an inter-kingdom HGT. From the maximum likelihood phylogenetic tree based on the curated seed alignment of PF00311 (Figure S1), we saw that plant PEPC is clustered with a group of PEPCs from gamma-proteobacteria,forming a sister group to other bacteria PEPCs. This phylogeny supported the idea that plant PEPCs is a lineage derived from ancestral bacteria PEPCs by means of an ancestral inter-kingdom HGT, contrary to the previous understandings that bacteria PEPCs originated from plant PEPCs. However, the plant PEPCs in the seed alignment all belong to the so-called BTPC group and many important eukaryotic taxa that are not plant, such as the protist and cellular slime mold, were not included in the seed alignment. To identify the origin of PTPC and PEPCs in the non-plant eukaryotic taxa, we carried out further phylogeny reconstruction of PEPCs from representative taxa in bacteria, archaea, plant and non-plant eukaryotes.

To explore the possible existence of inter-kingdom HGT in PEPC, we screened the full curation of PF00311 and PF14010 in the Pfam database to find inter-kingdom HGT candidates and included those candidates in the sequences for the following phylogenetic reconstruction. We searched the Pfam “full” tree to find the PEPC sequences from different kingdoms with the branches surrounding it. As no PEPC is found in fungi and only two are found in animals, we focused on divisions of the plants, bacteria and archaea. In total, we found 29 sequences from non-archaea organisms in the full tree of PF14010, 49 sequences from non-plant organisms and 30 sequences from non-bacteria organisms in the plant and bacteria divisions of the PF00311 full tree, respectively. Because the phylogeny of PF00311 contain 2976 sequences and many alignments of short fragments are represented on the tree and many internal branches have low bootstrap support value, we removed dubious candidates from short fragment of peptide (less than 300 amino acids), and used the remaining 21 sequences from non-plant organisms and 19 sequences from non-bacteria organisms to carry out further phylogenetic analysis.

Having collected the inter-kingdom HGT candidates from plant and bacteria, we carried out phylogeny reconstruction in combination with the sequences of the non-plant eukaryotic taxa, BTPC and PTPC from several plants and representative bacteria PEPCs curated in the seed alignment (Table 1). In total, we used 122 PEPCs for gene phylogeny reconstruction. For the inter-kingdom HGT in archaeaphylogenetic reconstruction, we used the sequences of all 77 members of PEPcase_2 and four bacteria PEPCs as outgroups. We first aligned the sequences and then adopted a program MUMSA to assess the quality in order to find the best alignment by calculating the multiple overlap score (MOS) that indicates the overall inter-consistency with other alignments (see Materials and Methods). The alignment with the highest MOS was selected as the best alignment, and those alignments were then used to carry out phylogeny reconstruction.

**Table 1:** Sequences used in the phylogenetic reconstruction.

*Taxon*	GenBank or Uniprot ID
***Acidimicrobium ferrooxidans***	256007505
***Acidobacterium capsulatum***	225874618
***Algoriphagus sp.***	311746515
***Arabidopsis thaliana g1***	15232442
***Arabidopsis thaliana g2***	30697740
***Arabidopsis thaliana g3***	240254631
***Arabidopsis thaliana g4***	15219272
***Arabidopsis thaliana g5***	222423984
***Archaeoglobus fulgidus***	11499081
***Aureococcus anophagefferens***	323453325
***Babesia bovis***	156084500
***Capsaspora owczarzaki***	320168251
***Chlamydomonas reinhardtii***	51701320
***Chlorobaculum parvum***	193085694
***Chloroflexus sp.***	222450523
***Cryptosporidium hominis***	67594757
***Cryptosporidium muris***	209881885
***Cryptosporidium parvum***	66357588
***Deinococcus deserti***	226355772
***Dictyoglomus thermophilum***	206740030
***Dictyostelium discoideum***	66806573
***Dictyostelium fasciculatum***	328865638
***Dictyostelium purpureum***	330798819
***Ectocarpus siliculosus***	299117425
***Emiliania huxleyi***	223670909
***Escherichia coli***	15804552
***Gemmatimonas aurantiaca***	226229154
***Haemophilus influenzae***	16273525
***Halobacterium sp.***	15791074
***Lentisphaera araneosa***	149200328
***Leptospira biflexa***	167780286
***Methanosarcina acetivorans***	229017561
***Methanothermobacter thermautotrophicus***	15678963
***Mycoplasma penetrans***	26554388
***Myxococcus xanthus***	108759396
***Nitrosomonas europaea***	30248603
***Oryza sativa g1***	222622510
***Oryza sativa g10***	115476100
***Oryza sativa g11***	15022444
***Oryza sativa g2***	51091643
***Oryza sativa g3***	222617602
***Oryza sativa g4***	115440043
***Oryza sativa g5***	115434082
***Oryza sativa g6***	115435200
***Oryza sativa g7***	50251800
***Oryza sativa g8***	9828445
***Oryza sativa g9***	222619275
***Phaeodactylum tricornutum g1***	219120583
***Phaeodactylum tricornutum g2***	327343197
***Physcomitrella patens g1***	168044057
***Physcomitrella patens g2***	168010333
***Physcomitrella patens g3***	168027443
***Physcomitrella patens g4***	168042979
***Physcomitrella patens g5***	168016115
***Physcomitrella patens g6***	168061648
***Picrophilus torridus***	48478036
***Pirellula staleyi***	283779027
***Plasmodium berghei***	68071185
***Plasmodium chabaudi***	70950271
***Plasmodium falciparum***	124808830
***Plasmodium knowlesi***	221060224
***Plasmodium vivax***	156102026
***Plasmodium yoelii***	83282693
***Polysphondylium pallidum***	281207688
***Pseudomonas aeruginosa***	347303632
***Pyrobaculum aerophilum***	18314050
***Pyrococcus furiosus***	18978347
***Rhodospirillum centenum***	209965727
***Selaginella moellendorffii g1***	302800171
***Selaginella moellendorffii g2***	302783266
***Selaginella moellendorffii g3***	302817036
***Selaginella moellendorffii g4***	302795803
***Streptobacillus moniliformis***	269123480
***Streptococcus thermophilus***	89143166
***Sulfolobus solfataricus***	15899028
***Synechococcus sp.***	87284805
***Thalassiosira pseudonana g1***	224000774
***Thalassiosira pseudonana g2***	223998678
***Verrucomicrobium spinosum***	171911854
***Vibrio cholerae***	227082762
***Volvox carteri g1***	302835908
***Volvox carteri g2***	302830816
***Halobacterium salinarum***	CAPPA HALSA (Q9HN43)
***Archaeoglobus fulgidus***	CAPPA ARCFU (O28786)
***Archaeoglobus veneficus***	F2KS60 ARCVE (F2KS60)
***Caldivirga maquilingensis***	CAPPA CALMQ (A8MBK0)
***Candidatus Caldiarchaeum***	E6N9G7 9ARCH (E6N9G7)
***Candidatus Kuenenia***	Q1PXR4 9BACT (Q1PXR4)
***Candidatus Methylomirabilis***	D5MHI6 9BACT (D5MHI6)
***Clostridium cellulovorans***	D9SUK0 CLOC7 (D9SUK0)
***Clostridium perfringens g1***	B1RBJ1 CLOPE (B1RBJ1)
***Clostridium perfringens g2***	B1BWT1 CLOPE (B1BWT1)
***Clostridium perfringens g3***	CAPPA CLOPE (Q8XLE8)
***Clostridium perfringens g4***	CAPPA CLOPS (Q0STS8)
***Clostridium perfringens g5***	B1RT70 CLOPE (B1RT70)
***Clostridium perfringens g6***	B1RJT6 CLOPE (B1RJT6)
***Clostridium perfringens g7***	CAPPA CLOP1 (Q0TRE4)
***Clostridium perfringens g8***	B1BFT5 CLOPE (B1BFT5)
***Clostridium perfringens g9***	B1V5L0 CLOPE (B1V5L0)
***Desulfonatronospira thiodismutans***	D6SP11 9DELT (D6SP11)
***Desulforudis audaxviator***	B1I2W1 DESAP (B1I2W1)
***Dictyoglomus thermophilum***	B5YCF7 DICT6 (B5YCF7)
***Ferroglobus placidus***	D3S0D1 FERPA (D3S0D1)
***Halobacterium salinarum***	CAPPA HALS3 (B0R7F9)
***Ignicoccus hospitalis***	A8A9C2 IGNH4 (A8A9C2)
***Ignisphaera aggregans***	E0SSB1 IGNAA (E0SSB1)
***Lactobacillus brevis***	C2D3X1 LACBR (C2D3X1)
***Lactobacillus buchneri***	C0WSM6 LACBU (C0WSM6)
***Lactobacillus hilgardii***	C0XL21 LACHI (C0XL21)
***Leptospirillum ferrodiazotrophum.***	C6HVN3 9BACT (C6HVN3)
***Leptospirillum rubarum.***	A3EQI3 9BACT (A3EQI3)
***Leptospirillum sp.***	B6AN75 9BACT (B6AN75)
***Leuconostoc citreum***	B1N089 LEUCK (B1N089)
***Leuconostoc gasicomitatum***	D8ME72 LEUGT (D8ME72)
***Leuconostoc kimchii***	D5T4D7 LEUKI (D5T4D7)
***Leuconostoc mesenteroides***	C2KKA6 LEUMC (C2KKA6)
***Leuconostoc mesenteroides***	CAPPA LEUMM (Q03VI7)
***Metallosphaera sedula***	CAPPA METS5 (A4YES9)
***Methanohalobium evestigatum***	D7E7Q5 METEZ (D7E7Q5)
***Methanoplanus petrolearius***	E1RII9 METP4 (E1RII9)
***Methanopyrus kandleri***	CAPPA METKA (Q8TYV1)
***Methanosarcina acetivorans***	CAPPA METAC (Q8TMG9)
***Methanosarcina barkeri***	CAPPA METBF (Q469A3)
***Methanosarcina mazei***	CAPPA METMA (Q8PS70)
***Methanospirillum hungatei***	CAPPA METHJ (Q2FLH1)
***Methanothermobacter marburgensis***	D9PXG9 METTM (D9PXG9)
***Methanothermobacter thermautotrophicus***	CAPPA METTH (O27026)
***Methanothermus fervidus***	E3GXT0 METFV (E3GXT0)
***Oenococcus oeni g1***	A0NKU8 OENOE (A0NKU8)
***Oenococcus oeni g2***	D3LBW5 OENOE (D3LBW5)
***Oenococcus oeni g3***	CAPPA OENOB (Q04D35)
***Picrophilus torridus***	CAPPA PICTO (Q6L0F3)
***Pyrobaculum aerophilum***	CAPPA PYRAE (Q8ZT64)
***Pyrobaculum arsenaticum***	CAPPA PYRAR (A4WJM7)
***Pyrobaculum calidifontis***	CAPPA PYRCJ (A3MVZ5)
***Pyrobaculum islandicum***	CAPPA PYRIL (A1RR50)
***Pyrococcus abyssi***	CAPPA PYRAB (Q9V2Q9)
***Pyrococcus furiosus***	CAPPA PYRFU (Q8TZL5)
***Pyrococcus horikoshii***	CAPPA PYRHO (O57764)
***Sulfolobus acidocaldarius***	CAPPA SULAC (Q4JCJ1)
***Sulfolobus islandicus g1***	CAPPA SULIA (C3N0D7)
***Sulfolobus islandicus g2***	CAPPA SULIY (C3N8C3)
***Sulfolobus islandicus g3***	CAPPA SULIL (C3MJE5)
***Sulfolobus islandicus g4***	F0NMR2 SULIH (F0NMR2)
***Sulfolobus islandicus g5***	CAPPA SULIN (C3NJA0)
***Sulfolobus islandicus g6***	CAPPA SULIM (C3MTS7)
***Sulfolobus islandicus g7***	D2PDY7 SULID (D2PDY7)
***Sulfolobus islandicus g8***	CAPPA SULIK (C4KJI5)
***Sulfolobus islandicus g9***	F0NG17 SULIR (F0NG17)
***Sulfolobus solfataricus g1***	CAPPA SULSO (Q97WG4)
***Sulfolobus solfataricus g2***	D0KUQ4 SULS9 (D0KUQ4)
***Sulfolobus tokodaii***	CAPPA SULTO (Q96YS2)
***Thermococcus barophilus***	F0LK16 THEBM (F0LK16)
***Thermococcus sibiricus***	C6A2T7 THESM (C6A2T7)
***Thermofilum pendens***	CAPPA THEPD (A1RZN3)
***Thermoproteus neutrophilus***	B1YBY2 THENV (B1YBY2)
***Thermoproteus uzoniensis g1***	F2L305 THEU7 (F2L305)
***Thermoproteus uzoniensis g2***	F2L5Y2 9CREN (F2L5Y2)
***Vulcanisaeta distributa***	E1QNA4 VULDI (E1QNA4)
***Acidobacterium capsulatum***	C1F4Y2 ACIC5 (C1F4Y2)
***Cellulomonas flavigena***	D5UGP1 CELFN (D5UGP1)
***Chitinophaga pinensis***	C7PRS5 CHIPD (C7PRS5)
***Dokdonia donghaensis***	A2TNK9 9FLAO (A2TNK9)
***Erythrobacter sp. g1***	A5P918 9SPHN (A5P918)
***Erythrobacter sp. g2***	A3WAI8 9SPHN (A3WAI8)
***Flavobacteria bacterium***	A3J3B3 9FLAO (A3J3B3)
***Flavobacteriales bacterium***	A8UJQ6 9FLAO (A8UJQ6)
***Flavobacterium johnsoniae***	A5FG47 FLAJ1 (A5FG47)
***Geobacter sp.***	B9M086 GEOSF (B9M086)
***Gramella forsetii***	A0M1G5 GRAFK (A0M1G5)
***Haladaptatus paucihalophilus***	E7QR15 9EURY (E7QR15)
***Halalkalicoccus jeotgali***	D8JA44 HALJB (D8JA44)
***Haloarcula marismortui***	Q5V4H5 HALMA (Q5V4H5)
***Haloferax volcanii***	D4GUG0 HALVD (D4GUG0)
***Halogeometricum borinquense***	E4NPR5 HALBP (E4NPR5)
***Halomicrobium mukohataei***	C7NYU1 HALMD (C7NYU1)
***Haloquadratum walsbyi***	Q18FG1 HALWD (Q18FG1)
***Halorhabdus utahensis***	C7NNW9 HALUD (C7NNW9)
***Halorubrum lacusprofundi***	B9LS13 HALLT (B9LS13)
***Haloterrigena turkmenica g1***	D2RVU2 HALTV (D2RVU2)
***Haloterrigena turkmenica g2***	D2S2A1 HALTV (D2S2A1)
***Haloterrigena turkmenica g3***	D2S1E1 HALTV (D2S1E1)
***Kordia algicida***	A9E081 9FLAO (A9E081)
***Kribbella flavida***	D2PKN1 KRIFD (D2PKN1)
***Leeuwenhoekiella blandensis***	A3XNY5 LEEBM (A3XNY5)
***Microbacterium sp.***	B1NEZ1 9MICO (B1NEZ1)
***Natrialba magadii***	D3SY20 NATMM (D3SY20)
***Physcomitrella patens***	A9SLH0 PHYPA (A9SLH0)
***Polaribacter irgensii***	A4BW74 9FLAO (A4BW74)
***Polaribacter sp.***	A2TXN6 9FLAO (A2TXN6)
***Populus trichocarpa***	B9PBR9 POPTR (B9PBR9)
***Ricinus communis***	B9T8D2 RICCO (B9T8D2)
***Riemerella anatipestifer g1***	E4T920 RIEAD (E4T920)
***Riemerella anatipestifer g2***	F0TPC5 RIEAR (F0TPC5)
***Riemerella anatipestifer g3***	E6JHS7 RIEAN (E6JHS7)
***Tetrahymena thermophila***	Q23YQ3 TETTH (Q23YQ3)
**uncultured haloarchaeon g1**	A5YSL4 9EURY (A5YSL4)
**uncultured haloarchaeon g2**	A7U0W6 9EURY (A7U0W6)
***Zunongwangia profunda***	D5BFE2 ZUNPS (D5BFE2)

**Figure 1:**
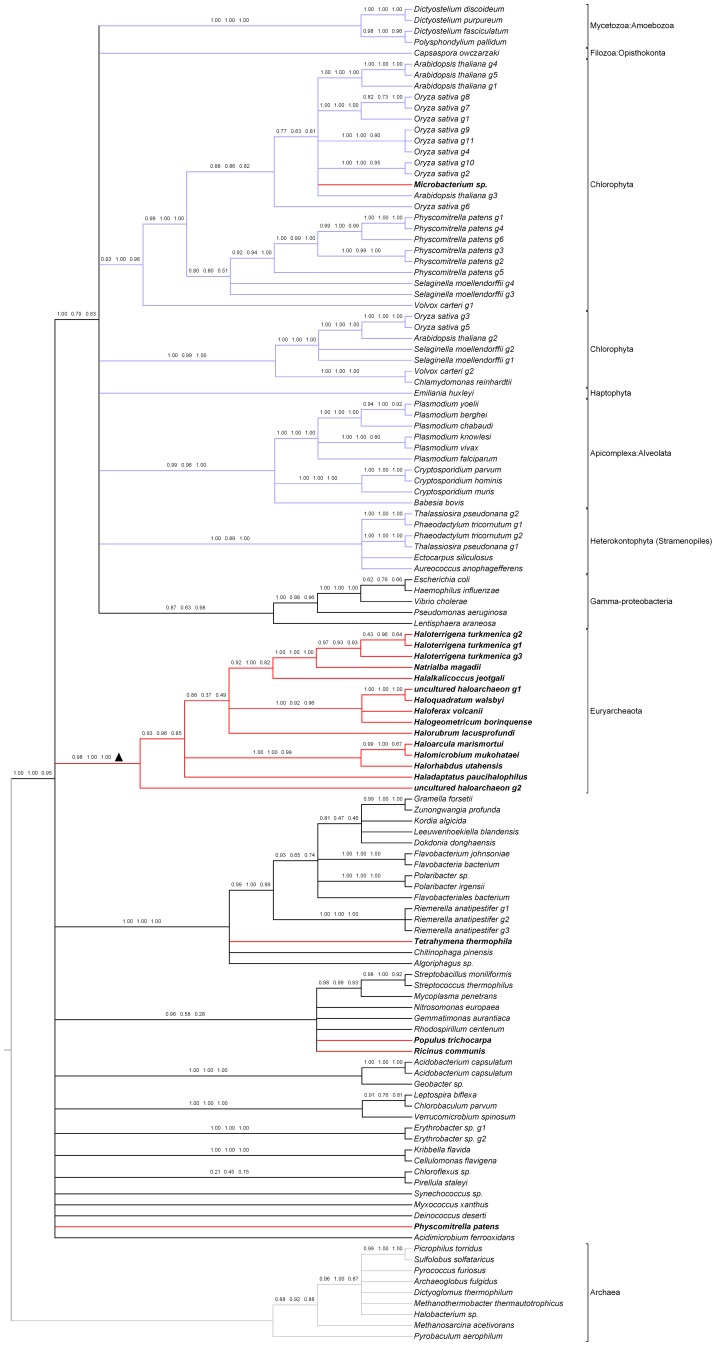
Phylogeny of bacteria and eukaryotic PEPcase and inter-kingdom HGT candidates. Phylogeny of inter-kingdom HGT candidates and PEPcase sequences from representative taxa in bacteria and eukaryotes were reconstructed. Nine archaea sequences were included as outgroups. HGT candidates confirmed in this phylogeny are in bold letters with red branches. The branches of outgrouparcheae are in grey and all eukaryotic branches are in blue. The bootstrap values of 100 replicates in the three different methods were labeled on each branch in order of maximum likelihood, neighbor joining and maximum parsimony. Ancient HGT events are marked with a triangle on the branch.

**Figure 2:**
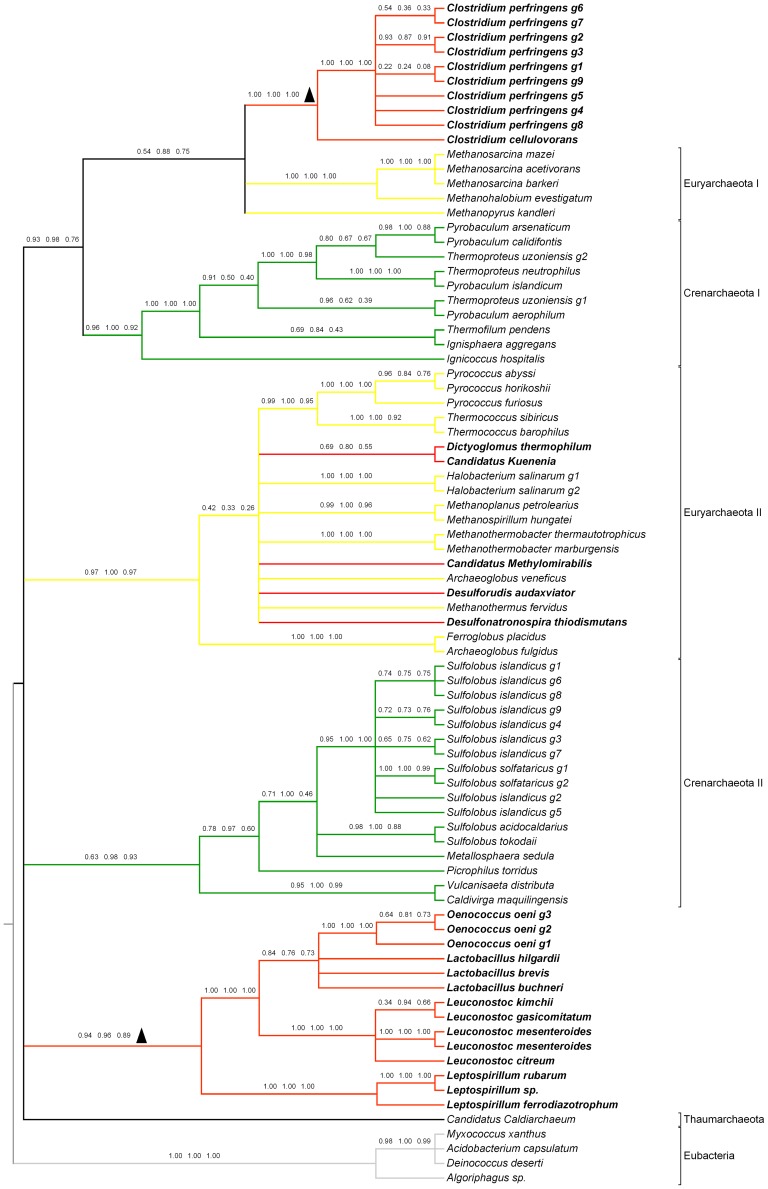
Phylogeny of archaea PEPcase and inter-kingdom HGT candidates. Phylogeny of PEPcase sequences from PF14010 were reconstructed. Four bacteria sequences were included as outgroups and their branches are in grey. HGT candidates confirmed in this phylogeny are in bold letters with red branches. The bootstrap values of 100 replicates are labeled in the same manner as Figure 1. Ancient HGT events were marked with triangles. Euryarchaeota branches were drawn in yellow while Crenarchaeota branches were in green.

We constructed the phylogenetic tree using three methods: maximum likelihood, neighbor joining and maximum parsimony. The protein substitution model used in maximum likelihood was selected by calculating the likelihood score under all 20 available models implemented in RAxML, and then we selected the model with the highest score. To avoid artificial resultscaused by improper construction methods, we combined the three trees to build a consensus tree that only contained branches supported by all the three methods. By inspecting this final consensus tree manually, we confirmed that there are 19 non-bacteria sequences clustered within the bacteria branches, a single non-plant sequence clustered within the plant branches (Figure 1) and 29 non-archaea sequences clustered within the archaea branches (Figure 2). To avoid artificial results due to uncertainty of alignment, we also repeated the phylogenetic analysis with the second best alignments and found no contradictory evidence (data not shown). To further exclude the possibility of artifacts due to alignment, we used GUIDANCE [9] to carry out alignment and bootstrap assessment of the alignment confidence and used only the high confidence columns (with bootstrap scores greater than 0.93) in the alignment to reconstruct the phylogeny. The results also showed no contradictory evidence with our major conclusion (See Figure S2 and S3). In Figure S2, the monophyly of all eukaryotic genes is supported by ML, NJ, MP with bootstrap value of 0.43, 0.64 and 0.17, respectively. However, the relation between eukaryotic groups (plant, protist, slime mold) is not consistent among three methods and most of the nodes are of low confidence. And for the pepcase_2 tree in Figure S3, the topology of NJ tree and MP tree are mostly consistent and those consensus nodes also receive high bootstrap support in NJ tree. The ML tree differs with the other two trees in the branch order of the basal branches. In the ML tree, thegroup of HGTs in Clostridium split first with the other archeae groups, while in NJ and MP trees a group of *Crenarchaeota* containing *Ignicoccushospitalis* diverges first with the other archeae groups. And also the NJ tree received the highest bootstrap support of those consensus nodes for pepcase_2. Compared with the computational cost of the ML and MP method, NJ seems to be the most efficient method among them.

And we also checked the genomic location of those candidates to exclude the possibility of sequence pollution for those un-clustered HGT genes. The result showed that most genes are from long genomic scaffolds except for the HGT genes in poplar and *Microbaterium* sp. which are from short fragments of 1,312 bp and 2,913 bp (Table S1). However, because the HGT gene in *Microbacterium* sp. clustered together with genes from seed plants and the possibility of genomic contamination of microbial genome library from multi-cellular organism is very low.We believe that the HGT in *Microbatierium* sp. is probably not the result of contamination. Further experiment is needed to exclude the possibility of genomic contamination for the HGT candidates in *Populustrichocarpa*. Collectively, in the evolution of phosphoenolpyruvate carboxylase gene family, we found 48 sequences originated from inter-kingdom HGTs. We also found that there three separate ancient HGT events,one from bacteria to archaea and the other two from archaea to bacteria,that respectively contributed to 15, 10 and 14 genes (Figure 1 and 2).

As for the origin of BTPC and PTPC, our phylogeny supported the idea that each type of PEPCs form a monophyletic group and both originated from ancestral bacteria lineage. That said, there is still uncertainty as to the precise relationship between these two groups and other eukaryotic PEPCs, due to inconsistency between different methods and low bootstrap support. This is consistent with the reality that the deep phylogeny of eukaryotes is still surrounded by controversy. Hopefully, further research on the basal phylogeny of eukaryotes will shed light on some of the controversy and further help explain the evolution of BTPC and PTPC. And our results also provide some information concerning the large scale phylogeny of the three life domains: Eukaryote, Eubacteria and Archeae. The well accepted phylogeny based on small-subunit (SSU) rDNA showed that Eukaryote and Archeae form a sister group with Eubacteria as the outgroup. However, many operational genes in Eukaryote are found to be more similar with homologs in Eubacteria while most eukaryotic informational genes is closer to their homologs in Archeae. And many hypothesis of symbiotic origin of Eukaryote are formed based on this finding. PEPC in Eukaryote is another gene originated via the horizontal gene transfer from bacteria symbiont (probably the ancestor of chloroplast) to the nucleus of the ancestral eukaryotic host [10], [11].

On a broader level, HGT was thought to be a relatively rare event in evolution. As more and more genome sequences become available, we continue to find many genes in the genome originated from HGT [12], [13], [14]. To date, however, there are no well-supported cases of multiple HGT events occurring in one gene family. One potential reason is that HGT was thought of as rare event, unlikely to hit a single gene family more than once. Consequently, little systematic research looking for HGT events in one gene family has been done. Our research provides the first case of multiple inter-kingdom HGTs in a single gene family and furthermore suggests that HGTsare much more frequent and important than previously expected. There is also research showing that HGT is more frequent between closely related organisms [15]. Here we opted to only look into the inter-kingdom HGT because HGTs between different kingdomsaremore readily identified when the intra-kingdom phylogeny of many species based on well recognized orthologs is not available. However, the frequency of all HGTs should be much higher than that of inter-kingdom HGT which we found in this study.

Successful HGTs involve two processes: the physical transfer of the genetic material into the recipient genome of another species, and the fixation of the gene in the population of the species by selection forces. Our findings are consistent with the fact that HGTs were found to be biased toward operational genes as opposed to informational gene because the operational gene can function and bring out fitness advantages with less interaction with other genes [11], [16]. PEPC is an operational gene that can function in many metabolic and developmental pathways but does not need many partner genes. We can only speculate that this may be the reason there are so many HGT events surrounding the evolution of this gene.

## Materials and Methods

We downloaded the protein sequences, alignment and phylogenetic trees of PEPcase (PF00311) and PEPcase_2 (PF14010) from the Pfam database [17]. Phylogenetic tree viewing and editing was done in the tree editor Archaeopteryx (0.960 beta A48) [18]. We cut the kingdom specific sub-trees for both bacteria and plant from Pfam full tree of PF00311. For archaea, we use the full Pfam tree of PF14010. Base on those kingdom specific tree, we use home-made scripts to find out the inter-kingdom HGT candidate, which is wrapped in the branches belong to a different kingdom in the Pfam tree. First, the taxonomy codes of all leaves were extracted from the sub-trees of bacteria, plant and archaea and searched in the UniProt taxonomy database [19]. We then inspected the taxonomy search results to find the taxa whose lineages do not contain the bacteria, plant or archaea. Finally, we extracted the full protein sequences and aligned fragments of those taxa from Pfam database; aligned fragments shorter than 300 amino acids were excluded from candidate list.

To validate the phylogenetic relationship between those HGT candidates and other members of PEPcase gene family and get a panorama of the gene family evolution in plant and bacteria, we collected the HGT candidates' full sequences and PEPcase sequences from representative taxa, totally 122 protein sequences to reconstruct the phylogeny of the gene family. For archaea, we used the full sequences of all PF14010 members. We applied four programs (T-Coffee, MAFFT, MUSCLE and ClustalW) to align the sequences and then assessed the quality of the alignments with Mumsa (online server at http://msa.sbc.su.se/cgi-bin/msa.cgi) [20], [21], [22], [23], [24]. All alignment programs were run using the default parameters, except T-Coffee where we used the “expresso” option.

The sequences in all alignments were sorted into the same order with MEGA5 [25] and then submitted to the Mumsa server to get the quality scores. Mumsa program calculates the MOS score of each alignment (See [24] for the detail of the algorithim). Briefly, the aligned residues shared by many alignments are more reliable, and the alignment with the largest number of such residues is supposed to be the closest to the true alignment [24]. We then selected the alignment with best quality to carry out phylogeny reconstruction with maximum likelihood, neighbor-joining and maximum parsimony methods. For maximum likelihood tree, we first use RAxML and a wrapperPERL script proteinmodelselection.pl to find the substitution model with highest likelihood score for the protein alignment, and then we used this substitution model with GAMMA model of rate heterogeneity and carried out rapid bootstrap test of 100 replicates [26]. The neighbor-joining tree was inferred using MEGA5 with distances calculated with Possion correction and bootstrap test of 100 replicates. The maximum parsimony tree was also inferred using MEGA5 with the Close-Neighbor-Interchange algorithm and bootstrap test of 100 replicates. We combined the consensus trees of three methods using TreeGraph2 and deleted the different methods' contradictory nodes [27]. Finally, inter-kingdom HGT genes were identified by manual inspection of the combined phylogenetic tree.

To further test our conclusion against alignment artifacts, we used the GUIDANCE webserver [9] to carry out alignment and assessment of the alignment accuracy. The analysis was carried out with default parameters, using MAFFT as the aligner and GUIDANCE as the algorithms for evaluating confidence scores, which measures the robustness of the alignment to guide-tree uncertainty. Then the high confidence columns of the alignments were extracted from the result with threshold of score 0.93. Then the filtered alignments were further used to reconstruct the phylogeny with three different methods (same as the above).

## Supporting Information

Figure S1 **Maximum likelihood tree of PF00311 seed alignment.** Phylogenetic tree of PF00311 seed alignment were downloaded from Pfam database and then midpoint-rooted and visualized with the tree viewer, Archaeoptertx 0.960 beta A48. All sequences were labeled in the Pfam style (UniProt protein ID+UniProt taxonomy ID+coordinates of beginning and ending of alignment). Bootstrap support values are labeled by the nodes. Plant PEPCs are marked with a curly bracket.
(PDF)

Figure S2 **Phylogeny of bacteria and eukaryotic PEPcase and inter-kingdom HGT candidatesbaced on filtered aligment with GUIDANCE.** Phylogeny of inter-kingdom HGT candidates and PEPcase sequences from representative taxa in bacteria and eukaryotes were reconstructed based on the filtered alignment result of GUIDANCE using three methods: a. Maximum Likelihood; b. Neighbor-Joining; c. Maximum Parsimony. Nine archaea sequences were included as outgroups. HGT candidates are in bold letters with red branches. The branches of outgrouparcheae are in grey and all eukaryotic branches are in blue. The bootstrap values of 100 replicate are labeled on the branches. The branch line widths were set with the support value.
(PDF)

Figure S3 **Phylogeny of archaeaPEPcase and inter-kingdom HGT candidatesbaced on filtered aligment with GUIDANCE.** Phylogeny of PEPcase sequences from PF14010 were reconstructed based on the filtered alignment result of GUIDANCE using three methods: a. Maximum Likelihood; b. Neighbor-Joining; c. Maximum Parsimony. Four bacteria sequences were included as outgroups and their branches are in grey. HGT candidates are in bold letters with red branches. The bootstrap values of 100 replicate are labeled on the branches. The branch line widths were set with the support value. Euryarchaeota branches were drawn in yellow while Crenarchaeota branches were in green.
(PDF)

Table S1 **Genomic information on singular HGT candidates.**
(DOCX)
