# Supplementary figures and images for: Multiple Inter-Kingdom Horizontal Gene Transfers in the Evolution of the Phosphoenolpyruvate Carboxylase Gene Family
Source: PLoS One. 2012 Dec 12;7(12):e51159. doi: 10.1371/journal.pone.0051159 (PMC3521007; doi:10.1371/journal.pone.0051159)

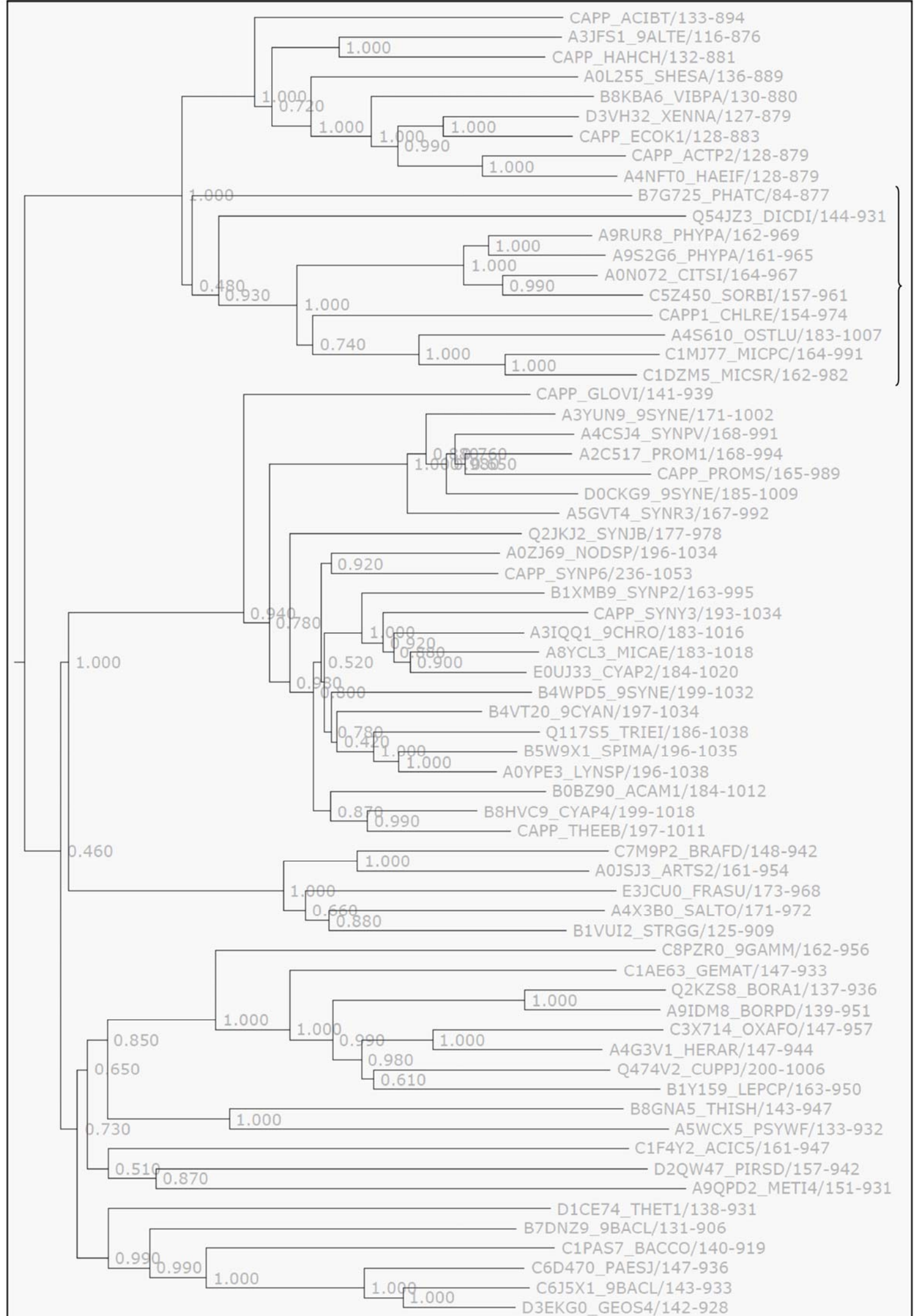

Supplement: Figure S1 — Maximum likelihood tree of PF00311 seed alignment. Phylogenetic tree of PF00311 seed alignment were downloaded from Pfam database and then midpoint-rooted and visualized with the tree viewer, Archaeoptertx 0.960 beta A48. All sequences were labeled in the Pfam style (UniProt protein ID+UniProt taxonomy ID+coordinates of beginning and ending of alignment). Bootstrap support values are labeled by the nodes. Plant PEPCs are marked with a curly bracket. (PDF) [file pone.0051159.s001.pdf]

Figure S2a

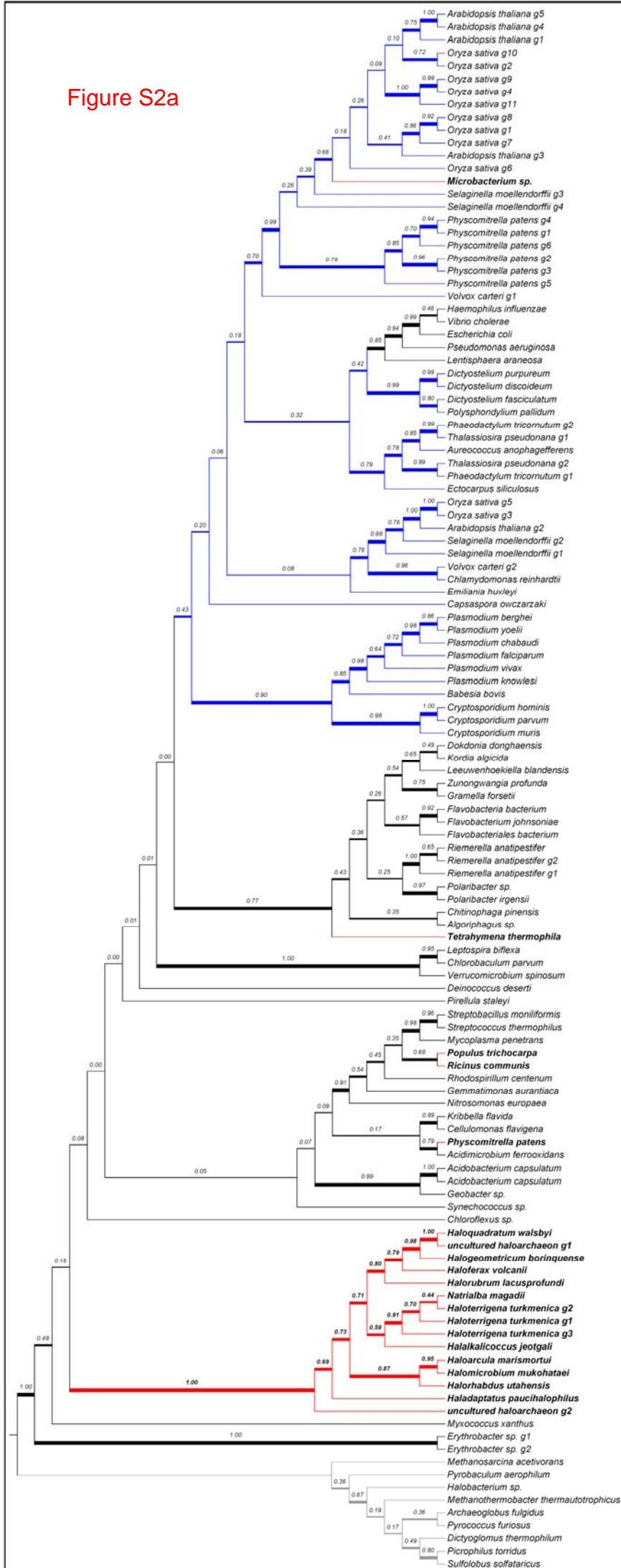

Figure S2b

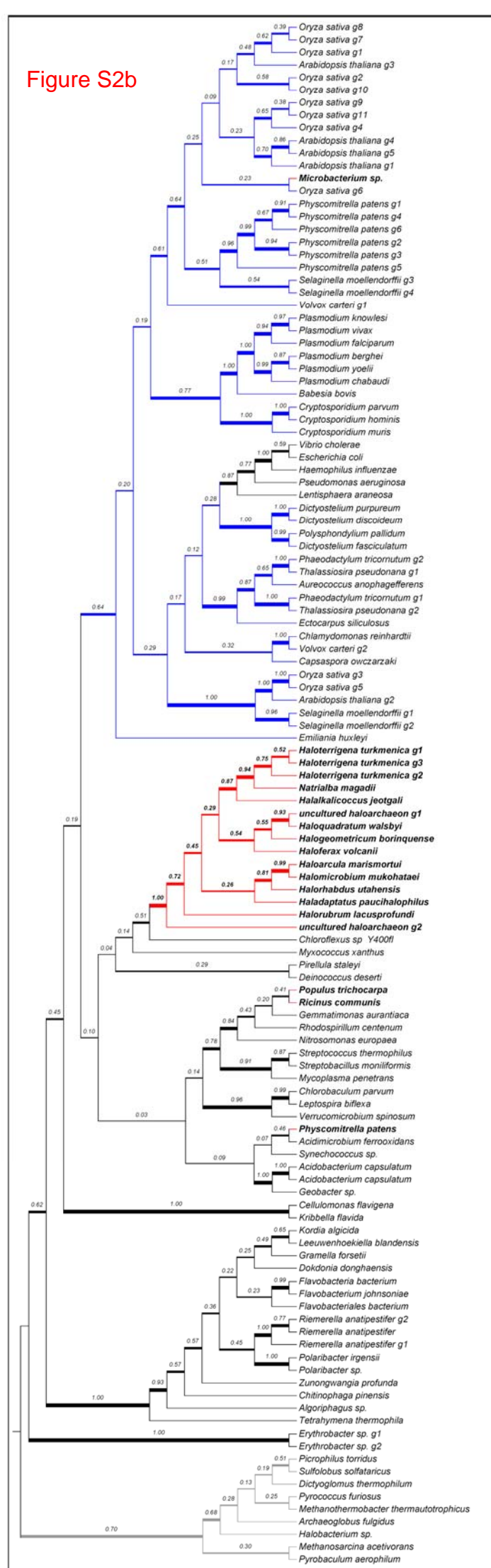

Figure S2c

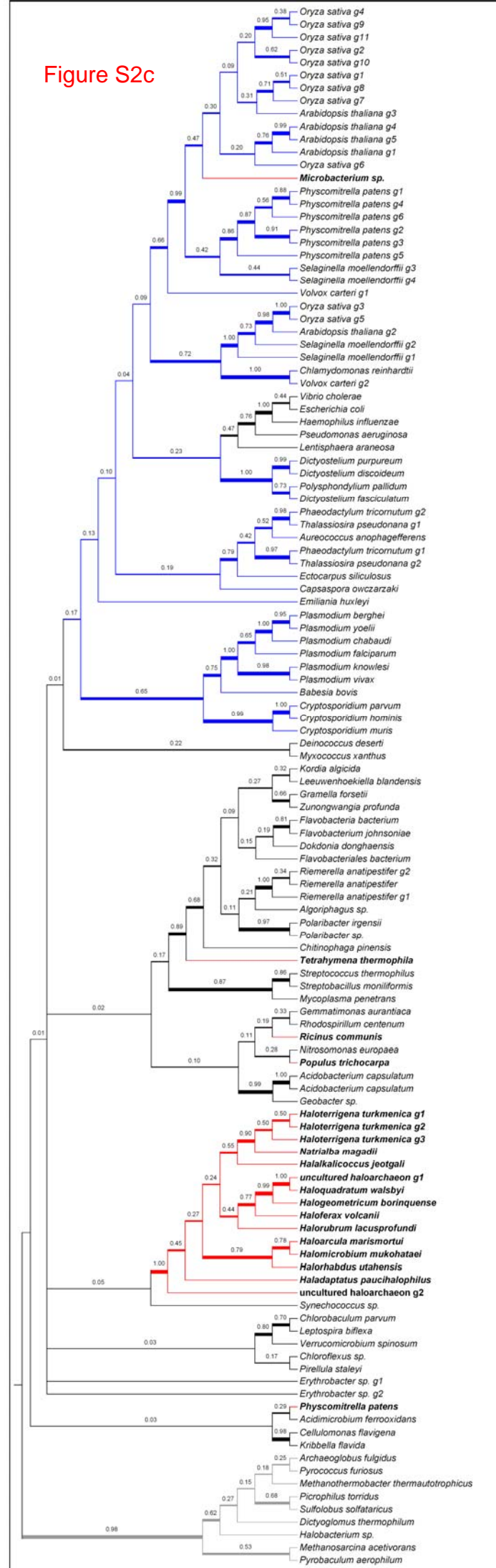

Supplement: Figure S2 — Phylogeny of bacteria and eukaryotic PEPcase and inter-kingdom HGT candidatesbaced on filtered aligment with GUIDANCE. Phylogeny of inter-kingdom HGT candidates and PEPcase sequences from representative taxa in bacteria and eukaryotes were reconstructed based on the filtered alignment result of GUIDANCE using three methods: a. Maximum Likelihood; b. Neighbor-Joining; c. Maximum Parsimony. Nine archaea sequences were included as outgroups. HGT candidates are in bold letters with red branches. The branches of outgrouparcheae are in grey and all eukaryotic branches are in blue. The bootstrap values of 100 replicate are labeled on the branches. The branch line widths were set with the support value. (PDF) [file pone.0051159.s002.pdf]

Figure S3a

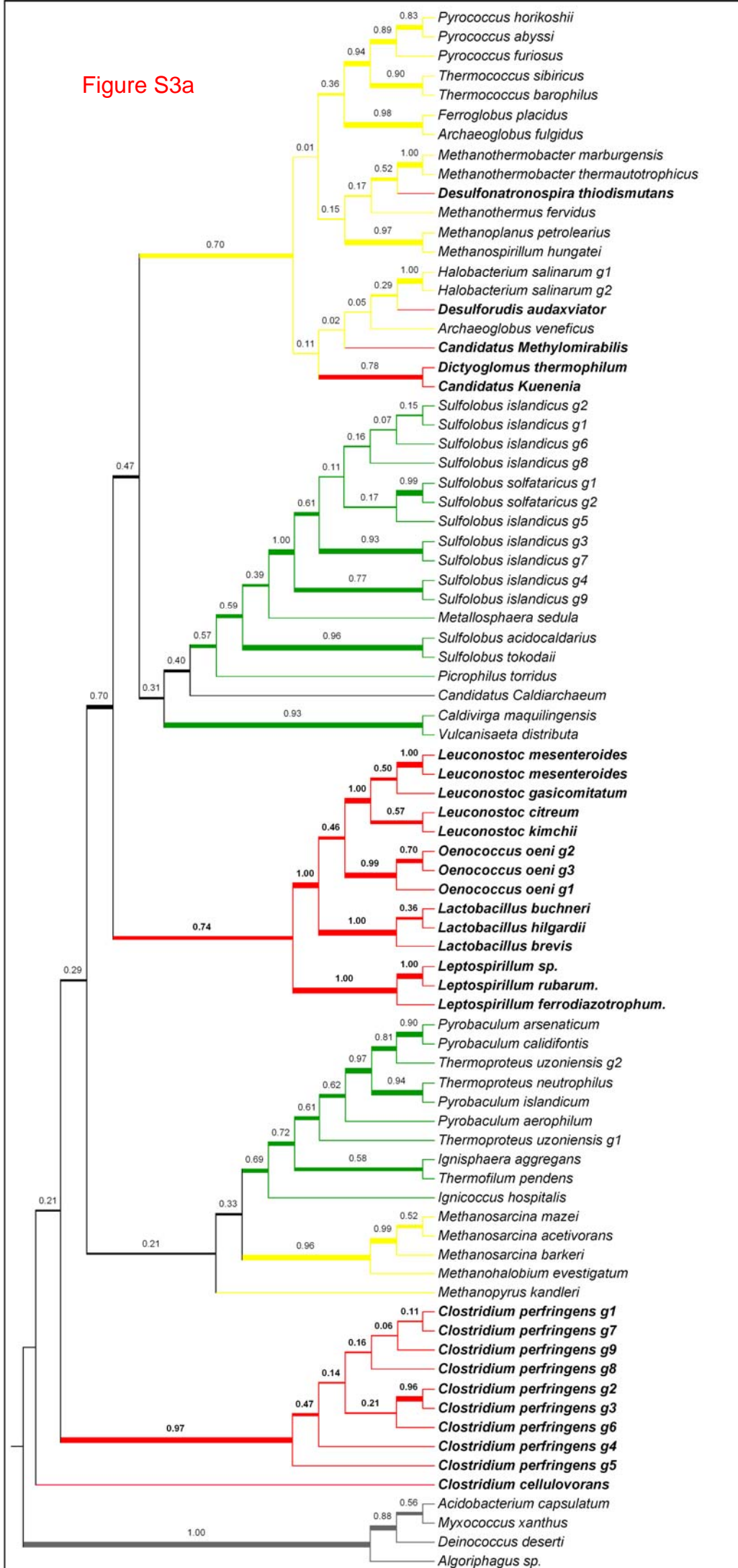

Figure S3b

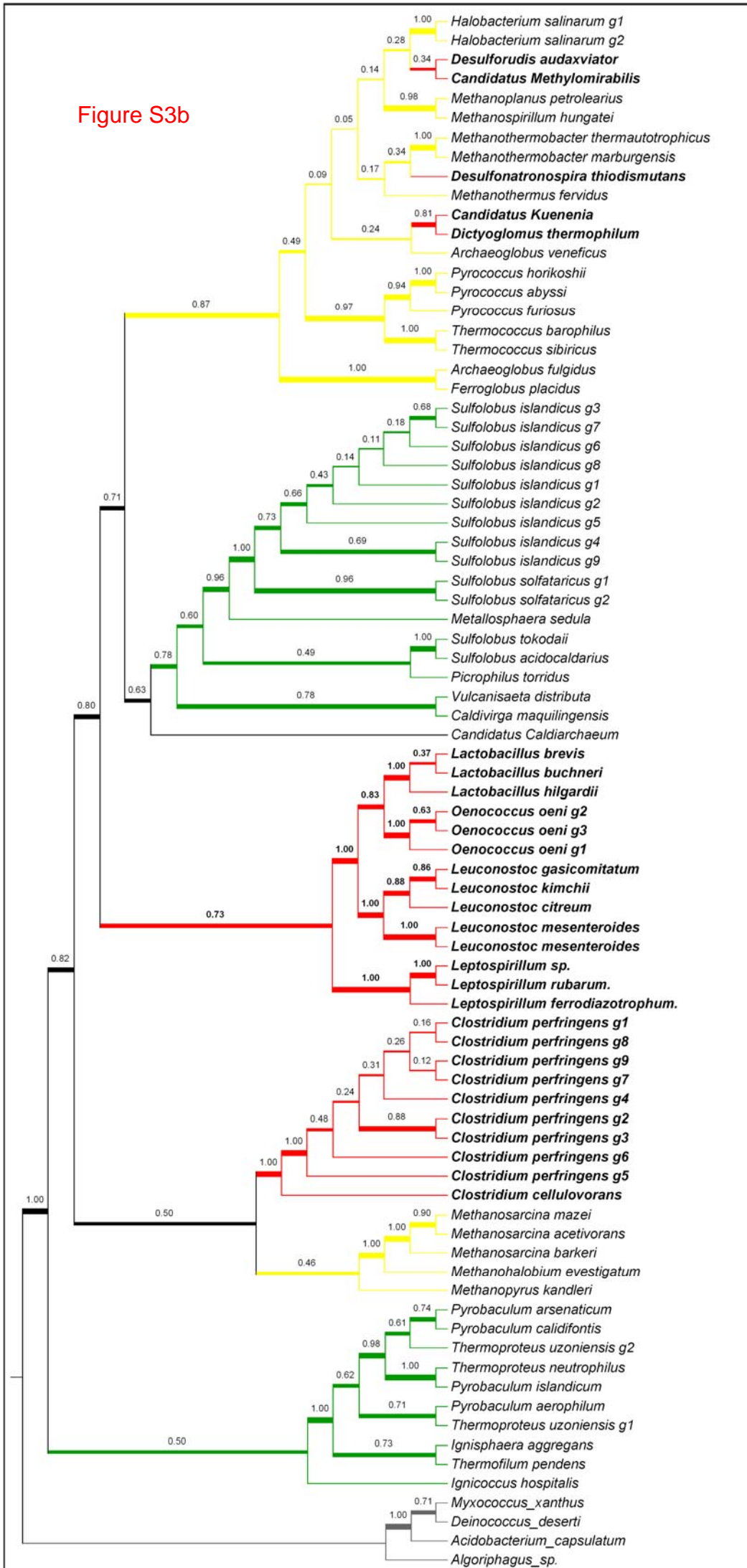

Figure S3c

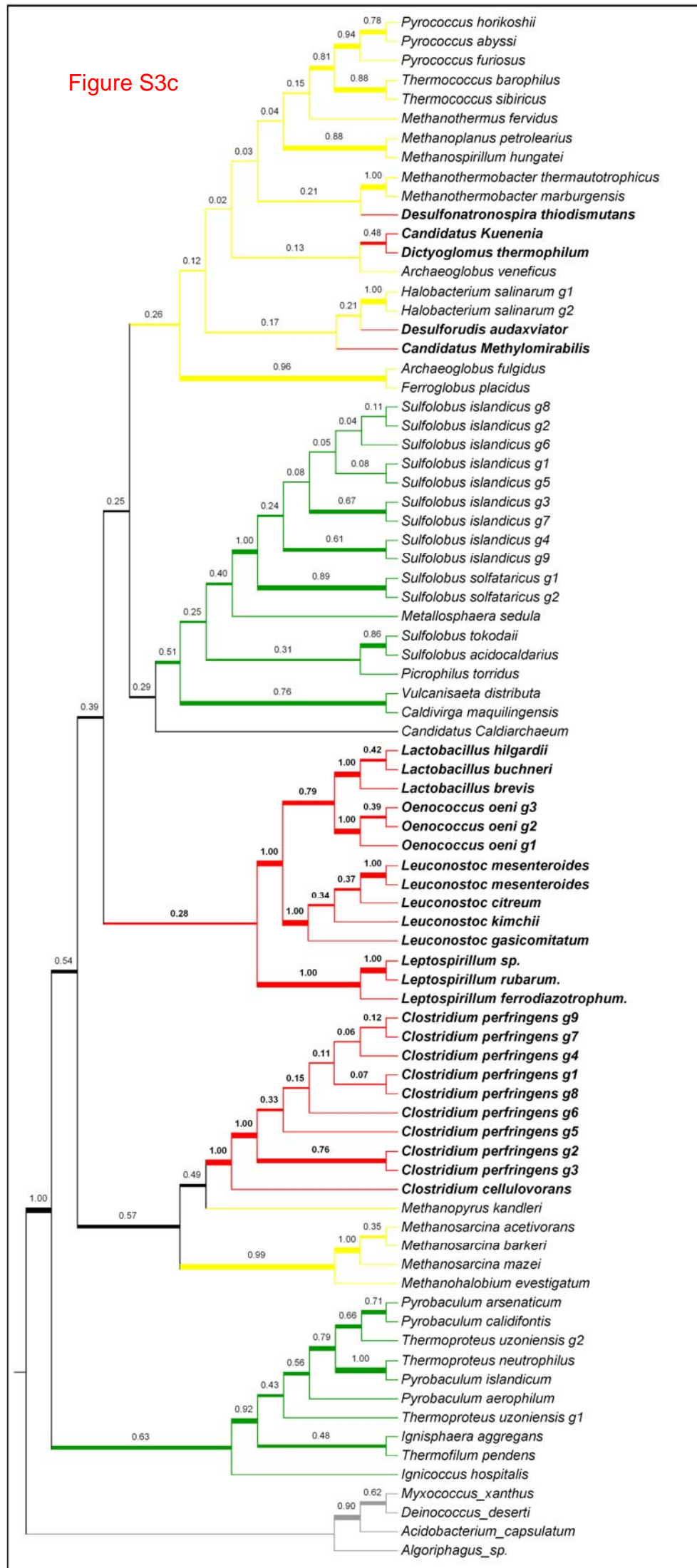

Supplement: Figure S3 — Phylogeny of archaeaPEPcase and inter-kingdom HGT candidatesbaced on filtered aligment with GUIDANCE. Phylogeny of PEPcase sequences from PF14010 were reconstructed based on the filtered alignment result of GUIDANCE using three methods: a. Maximum Likelihood; b. Neighbor-Joining; c. Maximum Parsimony. Four bacteria sequences were included as outgroups and their branches are in grey. HGT candidates are in bold letters with red branches. The bootstrap values of 100 replicate are labeled on the branches. The branch line widths were set with the support value. Euryarchaeota branches were drawn in yellow while Crenarchaeota branches were in green. (PDF) [file pone.0051159.s003.pdf]
