# Supplementary material for: Multiple Inter-Kingdom Horizontal Gene Transfers in the Evolution of the Phosphoenolpyruvate Carboxylase Gene Family
Source: PLoS One. 2012 Dec 12;7(12):e51159. doi: 10.1371/journal.pone.0051159 (PMC3521007; doi:10.1371/journal.pone.0051159)
Supplement: Table S1 — Genomic information on singular HGT candidates. (DOCX) [file pone.0051159.s004.docx]

**Table S1.** Genomic information on singular HGT candidates.

| **gene** | **genomic fragment** | **length (bp)** | **organism** |
| --- | --- | --- | --- |
| **Q23YQ3** | GG662558 | 69,572 | *Tetrahymena thermophila* |
| **B9PBR9** | EQ143916 | 1,312 | *Populus trichocarpa* |
| **B9T8D2** | EQ975004 | 17,979 | *Ricinus communis* |
| **A9SLH0** | DS544980.1 | 1,509,708 | *Physcomitrella patens* |
| **B1NEZ1** | EF449771 | 2,913 | *Microbacterium* sp. |
| **B5YCF7** | CP001146.1 | 1,959,987 | *Dictyoglomus thermophilum* |
| **Q1PXR4** | CT573072.1 | 896,449 | *Candidatus Kuenenia* |
| **D5MHI6** | FP565575.1 | 2,752,854 | *Candidatus Methylomirabilis* |
| **B1I2W1** | CP000860.1 | 2,349,476 | *Desulforudis audaxviator* |
| **D6SP11** | ACJN02000002 | 1,172,362 | *Desulfonatronospira thiodismutans* |
